# Diversity and community of methanogens in the large intestine of finishing pigs

**DOI:** 10.1186/s12866-019-1459-x

**Published:** 2019-04-29

**Authors:** Jiandui Mi, Haiyan Peng, Yinbao Wu, Yan Wang, Xindi Liao

**Affiliations:** 1 College of Animal Science, National Engineering Research Center for Breeding Swine Industry, South China Agricultural University, Wushan Road, Tianhe District, Guangzhou, 510642 People’s Republic of China; 2 Ministry of Agriculture Key Laboratory of Tropical Agricultural Environment, South China Agricultural University, Guangzhou, 510642 China; 3 Guangdong Provincial Key Lab of Agro-Animal Genomics and Molecular Breeding and Key Lab of Chicken Genetics, Breeding and Reproduction, Ministry of Agriculture, Guangzhou, 510642 China

**Keywords:** Methanogen, Redox potential, Large intestine, Pig

## Abstract

**Background:**

Methane emissions from pigs account for 10% of total methane production from livestock in China. Methane emissions not only contribute to global warming, as it has 25 times the global warming potential (GWP) of CO_2_, but also represent approximately 0.1~3.3% of digestive energy loss. Methanogens also play an important role in maintaining the balance of the gut microbiome. The large intestines are the main habitat for the microbiome in pigs. Thus, to better understand the mechanism of methane production and mitigation, generic-specific and physio-ecological characteristics (including redox potential (Eh), pH and volatile fatty acids (VFAs)) and methanogens in the large intestine of pig were studied in this paper. Thirty DLY finishing pigs with the same diet and feeding conditions were selected for this experiment.

**Result:**

A total of 219 clones were examined using the methyl coenzyme reductase subunit A gene (*mcrA*) and assigned to 43 operational taxonomic units (OTUs) based on a 97% species-level identity criterion. The family *Methanobacteriaceae* was the dominant methanogen in colonic digesta of finishing pigs, accounting for approximately 70.6% of the identified methanogens, and comprised mainly the genera *Methanobrevibacter* (57%) and *Methanosphaera* (14%). The order *Methanomassiliicoccales*, classified as an uncultured taxonomy, accounted for 15.07%. The methanogenic archaeon WGK1 and unclassified *Methanomicrobiales* belonging to the order of *Methanomicrobiales* accounted for 4.57 and 1.37%, respectively. The Eh was negative and within the range − 297.00~423.00 mV and the pH was within the range 5.04~6.97 in the large intestine. The populations of total methanogens and *Methanobacteriales* were stable in different parts of the large intestine according to real-time PCR.

**Conclusion:**

The major methanogen in the large intestine of finishing pigs was *Methanobrevibacter*. The seventh order *Methanomassiliicoccales* and species *Methanosphaera stadtmanae* present in the large intestine of pigs might contribute to the transfer of hydrogen and fewer methane emissions. The redox potential (Eh) was higher in the large intestine of finishing pigs, which had a positive correlation with the population of *Methanobacteriale*.

## Background

With the progression of global warming, research has been increasingly focused on greenhouse gas (GHG) emissions from livestock. This is due to the large number of livestock in the world and the rapid growth in the number of livestock in developing countries in recent decades [1, 2]. Methane has a global warming potential (GWP) 25 times that of CO_2,_ and represents substantial gross feed energy loss [3]. Although methane emissions from pigs are lower than those from ruminants, China farms the most pigs of any country in the world. In 2016, there were 0.435 billion pigs in China, accounting for 57% of the global total (data from National Bureau of Statistics of China). Methane emissions from pigs in China account for 10% of the total methane emissions from livestock [4]. The methane emissions from pigs also represent approximately 0.1~3.3% of digestive energy loss depending on the age and types of feed [5]. Therefore, reducing methane emissions from pigs is essential for controlling GHG emissions and improving feed efficiency.

Methane is produced by methanogens in the gut and manure, which mainly converts the substrates CO_2_ and H_2_ to methane [2]. Methanogens also play an important role in host health and have existed in the guts of pigs for millions of years [6, 7]. Some special methanogens exist in pigs; for example, *Methanobrevibacter gottschalkii* has been isolated from pig faeces [8]. Unlike the microbiota of ruminants, the microbiota of pigs, as monogastric animals, is mainly distributed in the hindgut [9]. Thus, to explore the mechanism of methane production, investigating the potential mitigation strategies from enteric fermentation, the mechanism of potential benefit for the host, the community composition and diversity of methanogenic archaea and the correlation with the parameters in the hindgut of pigs is essential. Methyl coenzyme Mreductase (*mcrA*) encodes that catalyses the terminal step in methane emissions and is ubiquitous among known methanogens [10]. Additionally, the relationship between *mcrA* transcription and methanogenesis has a positive correlation, meaning *mcrA* can be used as a biomarker for methanogenesis [11]. Some studies have investigated methanogens in the faeces of pigs or in vitro fermentation systems [12–14]. However, faeces and in vitro systems may not accurately represent the hindgut environment. Therefore, we investigated the diversity of methanogens in the hindgut of finishing pigs using an *mcrA* gene clone library and real-time PCR analysis. Because oxidation-reduction potential (Eh), pH and VFA production are regarded as the main factors affecting methanogen activity, these parameters were also determined to confirm the relationship between the gut environment and the diversity and community of methanogens in the hindgut of finishing pigs. Methanogens are an exclusively anaerobic microbiome that can only grow in low Eh environments. Otherwise, methanogens would be inhibited or unable to survive after oxygen exposure. Therefore, we will focus on the interaction between Eh and methanogens in this study.

## Results

### The diversity and community structure of methanogens in the hindgut of finishing pigs

**Fig. 1:**
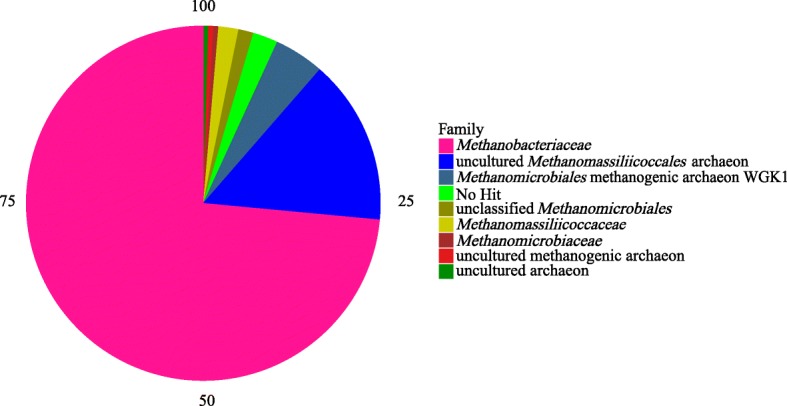
Taxonomic composition of methanogen (*mcrA*) communities from the clone libraries of finishing pigs

A total of 219 positive clones were obtained from the *mcrA* gene amplicons from the colonic digesta of finishing pigs (Table 1). The coverage of the library was 80%. The Chao1 index, Shannon index, and Simpson index of the library were 3.219, 97.6 and 0.077, respectively. Five sequences were not assigned to any methanogen taxa in the database (Table 1). The remaining 214 sequences were classified into 38 OTUs. Of these, 101 sequences belonged to *Methanobrevibacter* sp. WBY1. Of these, 26 and 24 sequences, were identified as *Methanosphaera stadtmanae* DSM 3091 and uncultured *Methanomassiliicoccales* archaeon, respectively. These three OTUs represented 70% of the valid sequences. The family *Methanobacteriaceae* was the dominant methanogen in the colonic digesta of finishing pigs, accounting for approximately 70.6%. *Methanobacteriaceae* mainly comprised the genera *Methanobrevibacter* (57%) and *Methanosphaera* (14%) (Table 1). The order *Methanomassiliicoccales* was identified as an uncultured taxonomy, accounting for 15.07% (Fig. 1). The methanogenic archaeon WGK1 and unclassified *Methanomicrobiales* belonging to the order of *Methanomicrobiales* accounted for 4.57 and 1.37%, respectively. We also identified the families *Methanomassiliicoccaceae*, *Methanobacteriaceae*, *Methanomassiliicoccaceae*, and *Methanomicrobiaceae* at less than 1%. At the species level, *Methanobrevibacter* sp. WBY1 (119 sequences), *Methanobrevibacter smithii* (2 sequences)*, Methanobrevibacter olleyae* (1 sequence)*,* and *Methanosphaera stadtmanae* DSM 3091 (31 sequences) were detected in the colonic digesta of finishing pigs (Table 1).

**Table 1:** Operational taxonomic units (OTUs) of *mcrA* gene sequences from colonic digesta of finishing pigs

OTU_*mcrA*_^b^	Clones	Nearest Taxon	% Sequence Identity
OTU0	1	NH^a^	\
OTU1	1	*Methanobrevibacter* sp. WBY1 (EU919429.1)	93
OTU2	1	NH	\
OTU3	2	Methanogenic archaeon WGK1 (GQ339874.1)	99
OTU4	1	Uncultured Archaeon (AB557213.1)	81
OTU5	1	*Methanosphaera stadtmanae* DSM 3091 (AJ584650.1)	85
OTU6	3	*Methanobrevibacter smithii* (CP017803.1)	95
OTU7	1	*Methanobrevibacter* sp. WBY1 (EU919429.1)	92
OTU8	2	*Methanosphaera stadtmanae* DSM 3091 (AJ584650.1)	84
OTU9	101	*Methanobrevibacter* sp. WBY1 (EU919429.1)	97
OTU10	1	*Methanobrevibacter olleyae* (CP014265.1)	92
OTU11	1	Candidatus *Methanoplasma termitum* (CP010070.1)	88
OTU12	2	Candidatus *Methanoplasma termitum* (CP010070.1)	84
OTU13	4	uncultured *Methanobrevibacter* sp. (JF973609.1)	94
OTU14	7	Methanogenic archaeon WGK1 (GQ339874.1)	85
OTU15	1	NH	\
OTU16	1	*Methanobrevibacter* sp. WBY1 (EU919429.1)	96
OTU17	1	uncultured *Methanomassiliicoccales* archaeon (KT225447.1)	82
OTU18	1	*Methanobrevibacter* sp. WBY1 (EU919429.1)	96
OTU19	1	*Methanobrevibacter* sp. WBY1 (EU919429.1)	92
OTU20	1	uncultured *Methanomassiliicoccales* archaeon (EF628111.1)	81
OTU21	3	unclassified *Methanomicrobiales* (miscellaneous) (GQ339874.1)	93
OTU22	2	*Methanosphaera stadtmanae* DSM 3091 (AJ584650.1)	84
OTU23	5	*Methanobrevibacter* sp. WBY1 (EU919429.1)	93
OTU24	1	uncultured *Methanobrevibacter* sp. (KC618377.1)	96
OTU25	1	Candidatus *Methanomethylophilus*. (KC412011.1)	97
OTU26	1	*Methanobrevibacter* sp. WBY1 (EU919429.1)	90
OTU27	1	*Methanobrevibacter* sp. WBY1 (EU919429.1)	84
OTU28	1	NH	\
OTU29	1	*Methanobrevibacter* sp. WBY1 (EU919429.1)	85
OTU30	1	uncultured *Methanoculleus* sp. (AM284387.1)	100
OTU31	1	*Methanobrevibacter smithii* (LT223564.1)	88
OTU32	6	uncultured *Methanomassiliicoccales* archaeon (KT225454.1)	86
OTU33	26	*Methanosphaera stadtmanae* DSM 3091 (AJ584650.1)	88
OTU34	1	NH	\
OTU35	1	*Methanobrevibacter* sp. WBY1 (EU919429.1)	93
OTU36	24	uncultured *Methanomassiliicoccales* archaeon (KT225454.1)	85
OTU37	1	*Methanobrevibacter* sp. WBY1 (EU919429.1)	96
OTU38	1	Methanogenic archaeon WGK1 (GQ339874.1)	94
OTU39	1	*Methanobrevibacter smithii* (CP017803.1)	84
OTU40	1	uncultured methanogenic archaeon (EF628097.1)	90
OTU41	1	*Methanobrevibacter* sp. WBY1 (EU919429.1)	88
OTU42	1	uncultured *Methanomassiliicoccales* archaeon (KT225454.1)	75
OTU43	2	*Methanobrevibacter* sp. WBY1 (EU919429.1)	94

^a^NH-No hit sequence on methanogens in the database

^b^OTU_*mcrA*_-*mcrA* sequences were obtained from the DOTUR program as a unique sequence, while OTUs were generated by the DOTUR program at 97% species-level identity

### Phylogeny of abundant methanogens

**Fig. 2:**
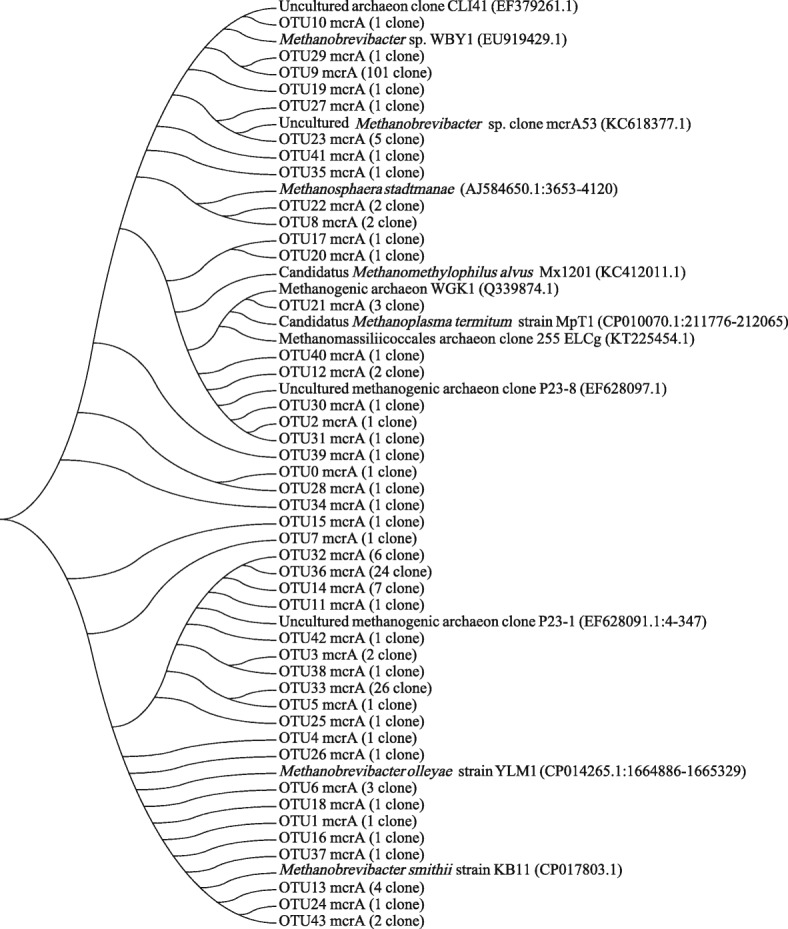
Phylogenetic analysis of methanogen partial *mcrA* sequences from finishing pig clone libraries inferred using MEGA (ver. 7). Evolutionary distances were calculated using the Neighbour-joining method. The tree was bootstrap resampled 1000 times.The 219 clones examined were assigned to 43 OTUs by DOTUR using a 97% species-level identity

To investigate the phylogenetic placement of OTUs_*mcrA*_ methanogen sequences from finishing pigs, the clone reference sequences were aligned to build a distance-matrix phylogenetic tree (Fig. 2). Most OTUs_*mcrA*_ clustered with *Methanobrevibacter* of different species (Fig. 2). A total of 91 OTUs_*mcrA*_ clustered closely with an unclassified sequence from the database and was not affiliated with any cultured species. Four OTUs_*mcrA*_ clustered with *Methanosphaera stadtmanae*. Three OTUs_*mcrA*_ were affiliated with Candidatus *Methanoplasma termitum*.

### The abundance of total methanogens and order *Methanobacteriales* and other parameters in the colonic samples

**Fig. 3:**
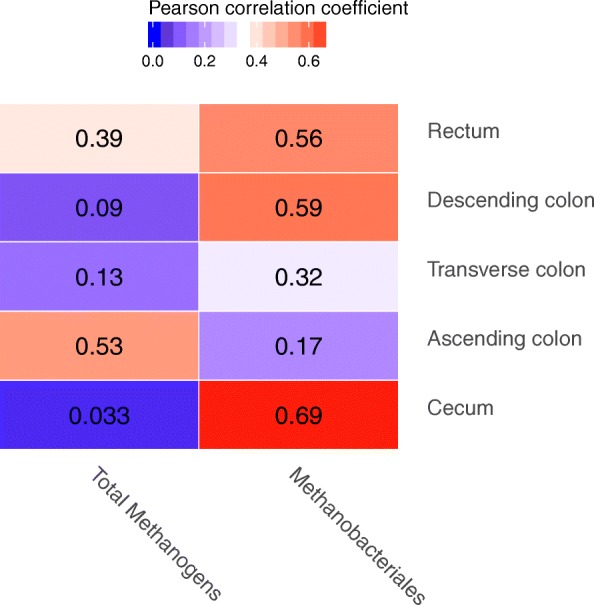
The relationship between the number of total methanogens, *Methanobacteriales* and Eh

The copy number of total methanogens and *Methanobacteriales* was not different in different gut intestines (Table 2). The Eh was negative and within the range of − 297.00~423.00 mV in the large intestine, showing an increasing trend from the caecum to the rectum in the digesta of the large intestine of finishing pigs. The pH was within the range of 5.04~6.97 in the large intestine. Both pH and total VFAs had no significant difference among different intestines of finishing pigs. The acetate and propionate levels were lowest in the digesta of the rectum (*P* < 0.05, Table 3). The correlation between the number of methanogens and Eh is shown in Fig. 3.

**Table 2:** The population Log 10 (copy number/μg DNA) of methanogens and *Methanobacteriales* in the different large intestines of finishing pigs

	Caecum	Ascending colon	Transverse colon	Descending colon	Rectum
Total Methanogens	7.81 ± 0.51	8.32 ± 0.33	8.22 ± 0.31	8.22 ± 0.21	7.75 ± 0.61
*Methanobacteriales*	5.83 ± 0.24	5.67 ± 0.17	5.86 ± 0.24	5.94 ± 0.31	6.30 ± 0.27

**Table 3:** The Eh (mV), pH and volatile fatty acid (VFA, mmol/L) values in the different large intestines of finishing pigs

Items	Caecum	Ascending colon	Transverse colon	Descending colon	Rectum
Eh	−379.47 ± 5.09^a^	−379.33 ± 3.63^a^	− 375.03 ± 4.19^a^	− 363.10 ± 6.52^ab^	− 355.50 ± 7.01^b^
pH	6.15 ± 0.08	6.11 ± 0.07	6.09 ± 0.07	6.10 ± 0.05	6.13 ± 0.06
Acetate	33.13 ± 1.65^ab^	33.50 ± 1.87^a^	29.43 ± 1.96^ab^	29.24 ± 1.69^ab^	27.82 ± 1.74^b^
Propionate	12.78 ± 0.99^a^	11.75 ± 0.91^b^	11.22 ± 1.00 ^b^	10.24 ± 1.01^b^	10.04 ± 0.98^b^
Butyrate	5.60 ± 0.74	7.26 ± 1.09	6.44 ± 0.90	5.19 ± 0.85	5.87 ± 0.73
A/P (Acetate to Propionate)	2.79 ± 0.12	3.19 ± 0.20	3.18 ± 0.38	3.49 ± 0.30	3.40 ± 0.34
Total VFAs	51.48 ± 3.03	52.41 ± 3.45	46.98 ± 3.41	44.57 ± 3.25	43.62 ± 3.01

Different letters in the same column indicate significant differences (*P* < 0.05)

## Discussion

The results of this study indicated that *Methanobrevibacter* was the dominant methanogen in the large intestine of finishing pigs. *Methanobrevibacter* mainly utilizes hydrogen and carbon dioxide to produce methane, which is similar to findings in ruminants [15]. Dietary fibre can increase *Methanobrevibacter* in the hindgut of pigs [13, 16, 17]. The hydrogen produced by bacteria is consumed by *Methanobrevibacter* and is beneficial for maintaining gut health by improving the degradation of fibre [18]. Similar results were found in our previous study of the hindgut in Lantang pigs [17]. All clone sequences belonged to *Methanobrevibacter* in the piglets fed with a basal diet [16]. *Methanobrevibacter* sp. WBY1 was the predominant methanogen (Table 1), followed by *M. smithii* and *M. olleyae*, in accordance with previous studies of pig faeces [12, 14, 16]. However, in our study, we did not find *M. gottschalkii*, which was isolated from pig faeces in a previous study [8]. Candidatus *Methanoplasma termitum* was observed in our study and recently divided into the seventh order of methanogens as *Methanomassiliicoccales*, previously designated *Methanoplasmatales* [19]. Unlike most methanogens that have a pathway for the reduction of CO_2_ to methyl coenzyme M, *Methanomassiliicoccales* produces methane by the reduction of methanol or methylamines, which contributes to lower methane emissions [19, 20]. A total of 29/219 OTUs*mcrA* belonged to *Methanosphaera stadtmanae* DSM 3091 in our study, which produce methane only by the reduction of methanol with H_2_ and acetate as a carbon source [21]. However, the sequence identity of *Methanosphaera stadtmanae* DSM 3091 and Candidatus *Methanoplasma termitum* was in the range of 84~88%, indicating that these methanogens in the hindgut of finishing pigs should be studied further.

Environmental parameters and VFAs are highly important for maintaining microbiome balance in the gut [22]. Eh is an important factor that influences the microbiome composition because oxidation-reduction reactions are needed by the microbiome [23]. Different microorganisms need specific Eh values to survive; in general, anaerobes require an Eh range from + 100 to − 250 mV [24]. Many studies have been conducted on ruminants [25, 26]. The Eh value varies mostly within the range from − 300 to + 200 mV in the digestive tract of ruminants, from − 130 to − 200 mV in the rumen medium and from − 145 to − 190 mV in the fluid of goats [27, 28]. The Eh values in the large intestine of finishing pigs in our study were from − 297 to − 423 mV and were lower than in rumen, indicating that the hindgut of finishing pigs has a stricter environment. The correlation analysis between Eh and *Methanobacteriales* shows that the higher Eh value within the range in our study improves the growth of *Methanobacteriales* in the rectum, the descending colon, and the caecum. Overall, the methanogens in pigs require stricter anaerobic conditions and are difficult to isolate and culture compared to those of ruminants. However, whether the high Eh values in the gut of finishing pigs improve the growth of methanogens requires further study. VFAs are generated via fermentation in the large intestine and maintain a pH between 6 and 7. In this study, the pH in the large intestine was between 5.04 and 6.97. The pH, total methanogens, and *Methanobacteriales* numbers were stable in the different large intestines. The reason for this result might be that the dominant effect of the identical diet for the finishing pigs in our study was the same.

Methanogens are more difficult to culture than bacteria. Metagenomics has been used to discover the dark matter of methanogen in the environment [29]. The 16S rRNA, mcrA or library clone sequences have similar compositions of methanogens [11, 16]. However, clone library is one of the classic methods to analyse the community of microorganisms. However, according to the results, the coverage of the library was only 80%, indicating an insufficient number of selected monoclones in our study. Recently, Koskinen et al. (2017) reported a new archaeal sequencing method to discover the specific archaeal communities associated with different sites in the human body [6]. This method could be used to investigate the methanogen diversity across different treatments with diet or age in pigs to improve the poor recovery of methanogen species in gut microbiome studies. Moreover, isolation and culture of a single methanogen to understand its generic-specific physio-ecological characteristics, and expanding the reference database for community analysis for sequencing are also necessary for CH_4_ emissions research [30]. However, cultivation of methanogens is difficult using traditional methods because of the strict conditions requirements. Culturomics, as a new technology, might contribute to addressing the difficulties of cultivation [31].

## Conclusions

The major methanogen in the large intestine of finishing pigs is *Methanobrevibacter*. The seventh order *Methanomassiliicoccales* and genus *Methanosphaera stadtmanae* in the large intestine of pigs might contribute to transferring hydrogen to reduce methane emissions. The redox potential (Eh) was high in the large intestine of finishing pigs and was positively correlated with the population of *Methanobacteriales*. New sequencing methods and culturomics should be used to expand the understanding of methanogens in the gut of pigs.

## Methods

### Animals and collection of samples

Thirty finishing pigs (Duroc * Landrace * Yorkshire), weighing 95 ± 5 kg (140–150 days old, of which half were male and half female) with the same diet and feeding conditions for 30 days, were selected for this experiment. The pigs were owned by Shenzhen Nongmu Meiyi Meat Industry Co., Ltd. Permission for using these pigs was granted by the senior management in the company. All experimental procedures involving animals were approved by the Animal Experimental Committee of South China Agricultural University (SYXK2014–0136). The composition and nutrient content of the experimental diets provided by the farm can be seen in Table 4. The pigs were slaughtered by stunning with electrical currents followed within 30 s with bloodletting. Bloodletting was completed within 5 min. All procedures followed the “operating procedures of pig slaughtering” (GB/T 17236–2008). Subsequently, the caecum, the ascending colon, the transverse colon, the descending colon, and the rectum were removed immediately. Eh, and pH were immediately measured with a 6010 ORP Analyzer (JENCO, USA) with an ORP electrode (Bowen, China) and an AZ8651 pH metre (Heng Xin, China). Approximately 10 g of digesta from each intestine was collected and placed immediately into liquid nitrogen and stored at − 80 °C for methanogen clone library construction and analysis, and VFA determination [32].

**Table 4:** Ingredients and composition of the diets of the finishing pigs

Ingredient, g per kg feed		Calculated composition	
Dry corn grain	690	Gross energy (MJ/kg)	13.39
Bean meal	200	NDF (mg/g) ^c^	162.3
Rapeseed meal	40	ADF (mg/g) ^d^	70.4
DDGS^a^	30	Crude protein (mg/g)	161
Premix^b^	40	Lysine (mg/g)	8.4
		Met + Cys (mg/g)	5.1
		Calcium (mg/g)	5.3
		Phosphorus (mg/g)	4.5
		Available phosphorus (mg/g)	1.9

^a^Distillers dried grains with solubles

^b^Commercial premix consisting of trace elements (i.e., Fe, Cu, Zn, Mn, I, and Se), vitamins (i.e., A, D, K, E, B1, B2, B6, B12, C, folic acid, and biotin), amino acids (i.e., lysine, and methionine), Ca, P and salts

^c^Neutral detergent fibre

^d^Acid detergent fibre

#### DNA extraction, clone library construction and DNA sequencing

DNA was extracted from 300 mg of wet colonic digesta using the bead-beating method followed by the Soil DNA kit (Omega, USA). The *mcrA* gene was amplified using primer pairs, and the amplification protocols were utilized according to previously report [33]. PCR products were purified using the EasyPure Quick Gel Extraction Kit (Trans, Beijing, China), ligated into pEASY-T3 (Trans, Beijing, Chain) and transformed into Trans1-T1 Phage resistant chemically competent cell. Plasmid DNA was recovered from recombinant cell colonies and the DNA library was screened by PCR analysis using previously described primer pairs [33]. A total of 219 positive insert-containing clones were randomly selected, and the nucleotide sequences of the clones’ inserts were determined by Beijing AuGCT DNA-SYN Biotechnology Co., Ltd.

#### Statistical analysis and phylogenetic analysis

The phylogenetic software package PHYLIP was used to calculate the evolutionary distances between pairs of nucleotide sequences [34]. The distance matrix was then used to assign nucleic acid segments in various OTUs using the furthest neighbour algorithm by DOTUR [35]. Nucleic acid sequences showing ≥97% identity were assigned to a similar OTU. The sampling effort in the library was evaluated by calculating the coverage (C) according to the eq. C = [1-(n/N)], where n is the number of sequences represented by a single clone and N is the total number of clones analysed in the library [36]. The Shannon index, Species Richness, and Simpson index were calculated by the SPADEprogram and were used to characterize species diversity in the library [37]. Sequences were compared with NCBI GenBank entries (https://www.ncbi.nlm.nih.gov) using the nucleotide–nucleotide BLAST. The phylogenetic tree was constructed by the neighbour-joining method of the MEGA 7 program using the bootstrap test based on 1000 replicates [38]. The sequences have been submitted to GenBank under the accession numbers JN105737-JN105780.

#### Real-time PCR analysis

The copy numbers of the 16S rDNA gene of the group-specific methanogens were quantified with SYBR Green real-time PCR analysis. All real-time PCR assays were performed using a LightCycler instrument (Mx3005P, USA). The characteristics of the primer sets for real-time PCR of methanogens and *Methanobacteriales* are listed in Table 5**.** Plasmid DNA of the target genes was extracted from positive recombinant plasmids and the DNA concentration was measured by Qubit 2.0 (ThermoFisher Scientific, USA). The serial gradient concentration of plasmid DNA was used to generate a standard curve for methanogens and *Methanobacteriales*. The copies of each target methanogen were run in triplicate, and the mean values were calculated using a standard curve.

**Table 5:** The characteristics of the primer and probe sets used in this study

Target group	Function	Sequence (5′-3′)	Tm (°C)	Amplicon Size (bp)	References
Methanogens	F primer	GGTGGTGTMGGATTCACACARTAYGCWACAG	58	470	Luton et al. (2002) [33]
	R primer	TTCATTGCRTAGTTWGGRTAGTT			
*Methanobacteriales*	F primer	GCCATGCACCWCCTCT	62	343	Yu et al. (2005) [39]
	R primer	TACCGTCGTCCACTCCTT			

## Abbreviations

ADF: Acid detergent fiber
DDGS: Distillers dried grains with solubles
GHG: Greenhouse gas
GWP: Global warming potential (GWP)
*mcrA*: methyl coenzyme mreductase
NCBI: National Center for Biotechnology Information
NDF: Neutral detergent fiber
OTUs: Operational taxonomic units
VFA: Volatile fatty acids
